# Bugs, drugs, and HIV: the role of the vaginal microbiome in HIV risk and antiretroviral efficacy for HIV prevention

**DOI:** 10.1186/s13073-017-0469-2

**Published:** 2017-08-17

**Authors:** Lenine J. P. Liebenberg, Derseree Archary, Aida Sivro, Douglas S. Kwon

**Affiliations:** 1 Centre for the AIDS Programme of Research in South Africa (CAPRISA), University of KwaZulu-Natal, Durban, South Africa; 2 Department of Medical Microbiology, University of KwaZulu-Natal, Durban, South Africa; 3 Ragon Institute of MGH, MIT, and Harvard, Harvard Medical School, Cambridge, MA USA; 4 Division of Infectious Diseases, Massachusetts General Hospital, Boston, MA USA

## Abstract

Advances in molecular tools to characterize the microbiome have led to the discovery of unique roles for microbes in human disease. Findings that the female genital microbiome can influence HIV acquisition and prevention emphasize the importance of microbiome analysis in clinical trials that assess the efficacy of HIV prevention interventions.

## Partial HIV protection demonstrated by the CAPRISA 004 vaginal microbicide

Globally, more than 90% of HIV is transmitted following heterosexual sex. Young women in sub-Saharan Africa represent a key target population for the development of new methods for HIV prevention, given that HIV prevalence is significantly higher in this population compared with young men [1]. In the absence of a vaccine or cure for HIV, emphasis has been placed on developing other biomedical tools that women can use to prevent HIV infection, such as vaginal microbicides, oral pre-exposure prophylaxis (PrEP), and better treatments for sexually transmitted infections. The Centre for the AIDS Programme of Research in South Africa (CAPRISA) 004 study was a double-blind, randomized, placebo-controlled trial conducted in KwaZulu-Natal, South Africa, which demonstrated that a single vaginal application of a microbicide gel containing the antiretroviral (ARV) tenofovir within 12 h before, and then after sex, reduced HIV acquisition by 39% overall, and by 54% in women with high levels of gel adherence [2]. Although poor adherence diminished drug efficacy in this and later microbicide trials [1], subsequent research identified genital inflammation [3] and the genital microbiome [4] as key biological factors that contributed to the efficacy of the gel. Thus, a deeper understanding of the causes of genital inflammation and the underlying mechanisms by which vaginal bacteria might lead to reduced efficacy of ARV-based strategies for PrEP is needed to produce effective HIV prevention modalities.

## Genital inflammation and HIV acquisition risk

During sexual transmission of HIV, productive infection is facilitated by micro-abrasions in the mucosal barrier and access to local activated CD4+ T cells (HIV target cells). Genital cytokine biomarkers of inflammation have been associated with both the number of local target cells and the potential to compromise the ability of the genital mucosa to serve as an effective barrier to HIV infection [5]. The combined effect of genital inflammation on mucosal barrier integrity and HIV target cell numbers likely contributes to the association of genital inflammation and infection by less-infectious HIV variants [6]. Recent findings have suggested that vaginal bacteria could be an important underlying contributor to baseline inflammation and HIV risk in women living in sub-Saharan Africa.

## The vaginal microbiome and HIV risk

Increasing evidence suggests that microbial communities in the female genital tract could directly impact genital inflammation and HIV acquisition risk. A study by Gosmann *et al*. [7] showed that young South African women with diverse vaginal microbial communities and low Lactobacillus abundance acquired HIV at over four-fold higher rates than those with *L. crispatus* dominance. Diverse vaginal microbial communities were closely associated with elevated genital inflammatory cytokines and cervical HIV target cells, which probably contributed to increased susceptibility to HIV. Furthermore, 90% of white women in developed countries have Lactobacillus-dominant vaginal communities [8], whereas the majority of South African women were found to have low Lactobacillus abundance without clinical indication of bacterial vaginosis, which highlights the importance of geography in defining what constitutes a ‘normal’ vaginal environment. Furthermore, behavioral, genetic and environmental factors are also likely to impact the effect of the vaginal microbiome on HIV risk. For example, the use of vaginal products for hygiene or for dry vaginal secretions to increase sexual pleasure has been reported to be common in several sub-Saharan African countries. These products might cause changes in vaginal pH and the microbiome, lead to increased inflammation, and disrupt the vaginal–epithelial barrier, which could impact HIV risk and microbicide efficacy in women [9].

## A role for Lactobacillus dominance in improving the efficacy of vaginal tenofovir gel

Recent findings by Klatt *et al*. [4] demonstrate that the vaginal microbiome can modulate the efficacy of topical tenofovir and suggests a novel mechanism for this relationship. Klatt *et al*. [4] utilized a metaproteomics approach to classify the host–microbial proteome of cervicovaginal lavage specimens from CAPRISA 004 participants. The authors identified two major bacterial communities based on relative microbial abundance: a Lactobacillus-dominant community, and a diverse, non-Lactobacillus-dominant community. Notably, the authors demonstrated that, in women with a Lactobacillus-dominant community, the risk of HIV acquisition was reduced by 61% using the tenofovir gel, whereas no significant HIV protection was observed in those with a vaginal microbiome dominated by non-Lactobacillus species. Furthermore, Klatt et al. [4] observed higher levels of genital tenofovir in women with Lactobacillus dominance than those who had non-Lactobacillus dominance, and attributed this to metabolism of tenofovir by *Gardnerella vaginalis* present in those with non-Lactobacillus-dominant communities. However, these observations might apply specifically to topical tenofovir-based microbicides, as bacterial vaginosis-associated bacteria appear to have no impact on the ability of oral tenofovir to protect women from HIV acquisition [10]. Future work is needed to better understand the specific bacterial community functions that potentially enable ARV metabolism. Additionally, a closer examination of the mechanism of host sensing of vaginal bacterial communities that lead to increased HIV acquisition risk might help identify novel targets to reduce this risk.

## Future considerations

Research on the relationship between the genital microbiome and HIV risk has raised several key considerations for future investigations. Klatt *et al*. [4] combined the use of proteomics and genomics platforms to classify vaginal bacterial communities in the absence of clinical measures of bacterial vaginosis such as the Nugent score. Despite good concordance between the proteomic footprint and 16S rRNA gene sequence data for some bacterial taxa, 16S rRNA analysis revealed diversity in the Lactobacillus-dominant group that the proteomics data could not resolve, which raises concerns about whether these approaches are adequate at this time to fully unravel the complexities of vaginal microbial communities. Further annotation of proteomic databases for the specific purposes of bacterial identification will help improve this approach and enable the simultaneous identification of downstream bacterial effectors. Complementary methodologies such as host single-cell transcriptomics, microbial metagenomics, and metabolomics will help assess the impact of the vaginal microbiome on genital inflammation, HIV risk, and PrEP efficacy. The Klatt *et al.* study [4] identified an in vitro impact of specific bacteria on drug metabolism, but the contribution in the context of a community of bacteria in the vagina is less established – as is the effect of various bacterial products on the mucosal environment. A further opportunity for investigation is longitudinal analysis of vaginal bacterial community dynamics, particularly given the influence of the menstrual cycle, endogenous hormones, hormonal contraceptives, and vaginal insertive practices.

Recent studies on the vaginal microbiome highlight the complexities of host–microbial interactions in the female genital tract and underscore the need for a deeper understanding of the interplay between the microbiome, host immune responses and bacterial metabolic function, and its impact on HIV acquisition risk. It will be important to understand whether the vaginal microbiome can be used to identify individuals at high risk of HIV acquisition or PrEP failure so that more-intensive measures to reduce risk can be taken. Ultimately, a deeper understanding of the vaginal microbiome might provide an opportunity to manipulate these communities to create durable alterations that decrease HIV acquisition risk, particularly in vulnerable women living in sub-Saharan Africa.

## Abbreviations

ARV: Antiretroviral
CAPRISA: Centre for the AIDS Programme of Research in South Africa
PrEP: Pre-exposure prophylaxis
